# Progress in Medicine

**Published:** 1899-09-09

**Authors:** 


### Progress in Medicine.

### FEVERS.

(*Continued from page* 367.)

Strasburger[1] reports a case of suppurative epididymitis following typhoid fever. In the first fortnight of convalescence the patient suffered from furuncles, gum-boils, and abscesses. In the third week an attack of epididymitis set in, the temperature rose to 100·5 deg. Two points of suppuration were detected and incised, the bacillus typhosus being detected in the evacuated pus. Ollivier has reported six such cases of abscess, and Curschmann six non-suppurative cases of epididymitis.

A severe epidemic of typhoid fever is at present existing in Philadelphia,[2] there having been 4,880 cases and 490 deaths between January 1st and March 24th. This condition is consequent upon a defective water supply, and during the past fifteen years repeated small outbreaks have given warning of the danger. The water which supplies the town is drawn from the Schuylkill River; it is highly polluted, unfiltered, and deficient in quantity. From the fact that the general death-rate is not increased in any notable degree, that children are chiefly attacked, and that the incidence of the disease is very unevenly distributed, it is evident that general sanitation is not satisfactory, and in part the outbreak is due to overcrowding and defective drainage. The death-rate from typhoid fever in Philadelphia is 60 per 100,000; that of towns supplied by pure mountain spring water, *e.g.*, Berlin and Munich, is 4; and of towns supplied from proper filter-beds, *e.g.*, London and the Hague, is 8·3 per 100,000. Abbott, of Philadelphia, the chief bacteriologist of the Board of Health, has, in consequence of the prevalence of disease in that city, been able in the past 20 months to test the serum reaction in 4,154 cases. The percentage of error has not been higher than 2·8. In reference to the serum reaction it may be mentioned that Piorkowski[3] claims that by isolating the bacillus typhosus from the stools by means of a special culture medium it is possible to diagnose the disease some days before Widal's reaction can be obtained. His medium consists of half per cent. peptone and 3·3 per cent. gelatine heated for one hour with normal urine, and filtered. The typhoid colony on such a surface presents a thread-like appearance, with threads radiating from the centre. Stab cultures are translucent, and do not spread over the surface.

The treatment of typhoid fever by means of subcutaneous injections of artificial serum has been investigated by Giglioli and Calvo.[4] Five hundred cubic centimetres of physiological solution of sodium chloride were injected into the outer aspect of the thigh. Whilst such treatment has no specific value it has a marked effect in relieving symptoms. In mild cases especially there is a feeling of relief, and a fall in temperature; in severe cases the effect is practically nil. The authors, therefore, suggest that as the injections are quite harmless they should be employed as a means of prognosis, since the intensity of the reaction varies directly as the intensity of the infection.

Moore,[5] whilst admitting the casual relationship existing between oysters and enteric fever, points out that following the eating of oysters may ensue an acute gastro-enteric catarrh, and, secondly, a specific continued fever, not typhoid, caused by ptomaïn poisoning. The gastro-enteritis happens within a few hours after unwholesome oysters are eaten; there is nausea, vomiting, purging, thirst, &c. The duration of the attack is a few hours only, and recovery is rapid. It is probably a simple irritation of the digestive track. If the poison be not eliminated by vomiting and purging, then poisonous albumoses are formed, and a few days after the toxic meal fever sets in. This may last from 7 to 14 days, and in fatal cases there may be peritonitis, convulsions, coma, and cardiac failure. All three stages of oyster poisoning may occur in one person, or of many persons exposed to infection one may suffer from gastro-enteritis, a second from ptomaïn poisoning, and a third from typhoid fever. An instance is given of an officer who suffered from an attack of gastro-enteritis after an oyster supper; this lasted two or three days. On the eighth day an anomalous continued fever developed, but soon passed off, and after several days of apyrexia enteric fever commenced.

**Cerebro-spinal Meningitis.**—This disease has during 1898 and 1899 been present in epidemic form in the United States. Troops in camp have been affected, and Class[6] reports an outbreak in Chicago. The mortality varies from 65 to 20 per cent., and is higher where sanitation is defective. The contagion is given off in the nasal and buccal secretions, but the specific organism outside the human body soon dies. Osler,[7] in the Cavendish Lecture for 1899, gives much valuable information regarding both the etiology and diagnosis of the disease. His remarks may be summarised as follows: Cerebro-spinal fever occurs in waves or periods. The average mortality is 68·5 per cent. In its history and course it is closely allied to pneumonia, but cerebro-spinal fever is caused by a specific organism, the diplococcus intracellularis. To render the diagnosis certain lumbar puncture should be performed, and from the fluid drawn off film preparations and plate cultures should be made. Lumbar puncture may afford temporary relief, but has no permanent therapeutic value. Clinically, the disease is characterised by abrupt onset, the irregular nature of the fever, and by the presence of rashes, erythema, petechiæ, herpes, and purpuric herpes. There is leucocytosis, but this is of no value for diagnostic purposes. Arthritis and periarthritis are not uncommon, and the diplococcus intracellularis has been isolated from the pus in the joints. A distinctive sign of this disorder is known as "Kernig's sign"; if the patient be seated up in bed and an attempt be made to extend the leg on the thigh there is a contraction of the flexors, preventing full straightening; but if the patient be placed in the recumbent position the leg can be fully extended. It appears probable that sporadic cases of the true cerebro-spinal meningitis have a low degree of infectivity, but the malignancy of the organism increasing it gives rise to an epidemic. Spinal meningitis complicating pneumonia is distinguished by the latency of spinal symptoms, by the presence of the lung lesion, and by its higher mortality. Treatment by drugs is of little avail. Morphine is the best drug for relieving pain. No good results have followed lumbar puncture or laminectomy.

**Malaria.**—Ross,[8] assuming that the part played by mosquitos in the spread of malaria is generally accepted, inquires: "What possibility is there of extirpating the disease?" That drainage and cultivation have beneficial effects is undoubted, but these measures cannot always be employed, nor can individual precautions, such as the use of punkahs and mosquito nets, be depended upon. He notes the existence of two genera of mosquitos, the Culex and Anopheles. In the latter only have malaria parasites been found, and they alone have been shown experimentally capable of transmitting the disease. Mosquitos of the Anopheles genus are to be found chiefly in the country. Their larvæ exist only in natural pools and puddles, never in domestic vessels, in artificial waters, or in pools or streams containing fish, or in small pools liable to be quickly dried up or scoured out by heavy rainfall. The methods to be followed are then obvious: (1) Ascertain by dissecting the mosquitos of the district whether malaria parasites are present; (2) detect their breeding grounds by examining the pools for larvæ; (3) fill up and drain away the pools, or if wells be affected medicate the water so as to kill the larvæ. Finlay,[9] who was in charge of troops in the neighbourhood of Santiago during the Cuban campaign, noted that although there were no mosquitos malaria was rife. This he attributed to the conveyance of the parasite by flies. He is a strong supporter of the theory of the spread of both malaria and yellow fever by means of the mosquito, believing that not only may a mosquito which has sucked blood from an infected person transmit disease by means of the salivary and venom glands, but that the eggs may be infected, and the new generation be capable of spreading infection. He recommends that all houses be provided with mosquito blinds, that larvæ should be destroyed by treating ponds, pools, &c., with permanganate of potash, that excreta be disinfected, and that sufferers from malaria and yellow fever be carefully guarded against the visits of mosquitos and other insects.

The question whether blackwater fever is a specific disease or a variety of malarial fever has given rise to much difference of opinion. Koch, in his recently-published Reese-Berichte, maintained that the condition described as blackwater fever was simply due to quinine poisoning, and was led by his experience in East Africa to believe that it was only seen as a manifestation of tertian malaria. Plehn found the malaria parasite in the blood of 17 out of 40 cases of blackwater fever; and Thin,[10] from the microscopic examination of organs of persons dying from this disease, comes to the same conclusion, and states that there is absolutely no evidence for the supposition that blackwater fever is due to a special form of parasite, but rather to an alteration in the degree of virulence. Ritchie[11] thinks that many of the cases described are in reality cases of yellow fever, and the remainder of severe malarial fever. Crosse[12] denies that the symptoms of the disease are due to quinine poisoning, and thinks that they are not yet in a position to speak authoritatively as to the etiology of the disease.

[1] Münch Med. Woch., Jan. 3, 1899. [2] Lancet, April 8, 1899. [3] Berlin Klin. Woch., Feb. 13, 1899. [4] Epitome, Brit. Med. Jour., Mar. 11, 1899. [5] Practitioner, Mar., 1899. [6] Med. Times, Feb., 1899. [7] Brit. Med. Jour., June 24, 1899. [8] Brit. Med. Jour., July 1, 1899. [9] Med. Rec., May 27, 1899. [10] Glas. Med. Jour., May, 1899. [11] Brit. Med. Jour., Mar. 25 et seq. [12] Lancet, Mar. 25, 1899.

